# A modular assembly cloning technique (aided by the BIOF software tool) for seamless and error-free assembly of long DNA fragments

**DOI:** 10.1186/1756-0500-5-303

**Published:** 2012-06-18

**Authors:** Nadezhda A Orlova, Alexandre V Orlov, Ivan I Vorobiev

**Affiliations:** 1 Laboratory of Biocatalysis, Institute of Bioorganic Chemistry, Russian Academy of Sciences, Miklukho-Maklaya 16/10, Moscow 119971, Russia; 2 “Datanium” Jsc, Lazorevyj proezd, 3 Bld. 5, Moscow 129323, Russia

**Keywords:** Molecular cloning, Software tool, Cloning technique, Hybrid restriction site

## Abstract

**Background:**

Molecular cloning of DNA fragments >5 kbp is still a complex task. When no genomic DNA library is available for the species of interest, and direct PCR amplification of the desired DNA fragment is unsuccessful or results in an incorrect sequence, molecular cloning of a PCR-amplified region of the target sequence and assembly of the cloned parts by restriction and ligation is an option. Assembled components of such DNA fragments can be connected together by ligating the compatible overhangs produced by different restriction endonucleases. However, designing the corresponding cloning scheme can be a complex task that requires a software tool to generate a list of potential connection sites.

**Findings:**

The BIOF program presented here analyzes DNA fragments for all available restriction enzymes and provides a list of potential sites for ligation of DNA fragments with compatible overhangs. The cloning scheme, which is called modular assembly cloning (MAC), is aided by the BIOF program. MAC was tested on a practical dataset, namely, two non-coding fragments of the translation elongation factor 1 alpha gene from Chinese hamster ovary cells. The individual fragment lengths exceeded 5 kbp, and direct PCR amplification produced no amplicons. However, separation of the target fragments into smaller regions, with downstream assembly of the cloned modules, resulted in both target DNA fragments being obtained with few subsequent steps.

**Conclusions:**

Implementation of the MAC software tool and the experimental approach adopted here has great potential for simplifying the molecular cloning of long DNA fragments. This approach may be used to generate long artificial DNA fragments such as *in vitro* spliced cDNAs.

## Background

A significant amount of sequence data for various organisms has accumulated in databases; however, not all of the information on the genes of interest to researchers is accurate [1,2]. At the same time, researchers are faced with the necessity of cloning the particular genes or non-coding genomic regions to establish or verify their functional role. Availability of sequence information makes complete genomic DNA library creation [3] unnecessary, thus speeding up research, since even advanced of genomic libraries creation techniques [4] are time-consuming.

However, working with genes that have not been fully investigated is risky because GenBank [5] entries can contain incorrect sequences. GenBank sequence accuracy and annotation problems have been widely discussed, for example, Wesche et al. [6] reported 0.1–0.2% mismatches in sequences of murine origin.

For our own research we needed to clone long genomic DNA sequences, but only partial sequence data (and unverified sequences for a closely related species) were available.

**Figure 1:**
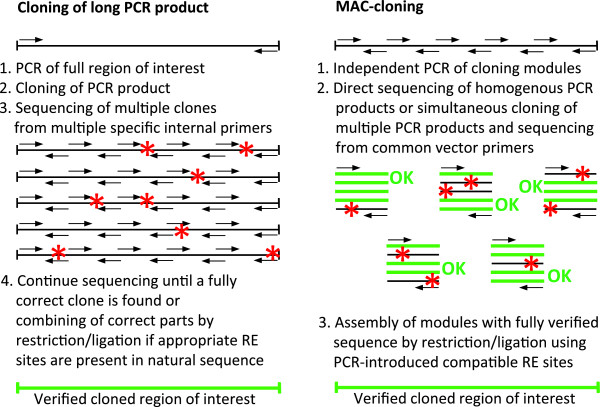
**Representation of the modular assembly cloning procedure.** Mutations or uncertainties are indicated as red asterisks. Sequence verified DNA is shown in green. Sequencing primers are represented by arrows.

The traditional approach for this type of work involves amplification of the target region using a high-fidelity thermostable DNA polymerase, which usually requires optimization of the PCR conditions, but often results in a small PCR product yield owing to the low processivity of high-fidelity DNA polymerases. Moreover, in practice, the error rates can be rather high, even for proof-reading polymerases. Experimental measurement of the mutation rate in PCR products showed that for a 349-bp fragment amplified by 30 PCR cycles, approximately 1% of clones had incorrect sequences [7]. It should be noted, that a significant proportion of PCR-introduced point mutations were not detected in the study because a functional forward mutation assay was used [7]. Additionally, when higher numbers of PCR cycles are used to amplify target fragments from vertebrate genomic DNA or large target fragment sizes, this can increase the level of incorrect PCR products to tens of per cents, making identification of non-mutated clones problematic. The search for correct PCR-generated long DNA fragments is further complicated by the need for multiple specific sequencing primers directed to various regions of the target DNA sequence instead of generic vector-specific primers with known performances (Figure 1).

In some cases, if a region of interest has a complex spatial structure and a non-optimal GC-content, optimization of the PCR conditions to obtain a long PCR product can become a challenging task. A number of PCR additives are commonly used for such cases (e.g. dimethyl sulfoxide [DMSO], betaine-Na, or alteration of Mg^+2^ ion concentrations), but they are not guaranteed to help, and may instead reduce the accuracy of the PCR. Moreover, if a PCR error is introduced at an early cycle, the option of sequencing more clones will not increase the reliability of the sequence data and the probability of picking a plasmid clone with a fully correct sequence will be marginal.

To overcome these limitations, we have developed an experimental approach and a computer tool which together simplify the process of generating a plasmid clone containing a long DNA fragment with an accurate sequence. The proposed modular assembly cloning (MAC) strategy is an alternative to direct PCR amplification and cloning for DNA fragments exceeding 3 kbp. Instead of attempts to obtain a sufficient yield of target long amplicon followed by extensive screening of clones with a low prospect of obtaining a clone without point mutations, the target fragment is divided into 500–1000-bp modules, or consecutive sub-fragments, each starting and ending with restriction sites and/or hybrid sites, generated by pairs of compatible restriction endonucleases (REs). An example of a compatible RE pair is *Nco*I and *Pci*I, which recognize CCATGG and ACATGT sites, respectively, producing compatible cohesive ends with CATG overhangs and a non-palindromic (hybrid) site (CCATGT) after ligation of the cut DNA fragments.

PCR-amplified modules are cloned individually into a plasmid vector; the sequence is verified using generic primers and assembled by sticky-end ligation of isolated inserts using the first cloned module (Figure 1). PCR products may also be added to the assembly without sub-cloning if their direct sequencing gives clear and satisfactory results. Because the restriction-ligation procedure rarely generates point mutations, re-sequencing of the assembled fragment is unnecessary. The MAC strategy minimizes the number of custom synthetic oligonucleotides for DNA sequencing, the number of sequencing runs, and allows rapid molecular cloning of long DNA fragments with a high level of accuracy.

The simplest way to divide a target DNA into modules is to identify the recognition sites of all of the available restriction enzymes that produce 4-nt overhangs. If we assume that the distribution of nucleotides in the DNA fragment is random, certain 6-nt sequences will occur once in every 4^6^ bp (4 096). There are 4^3^ (64) different 6-bp palindromes, therefore, one of all possible 6-nt palindromes should occur once in 4^3^ (64) nt. This number of RE recognition sites appears to be sufficient for finding at least one suitable site within a 100–200 bp area, but a significant proportion of REs will have no recognition sites in the entire target DNA fragment (the probability of an absence of recognition sites within a 5000-bp fragment for a given 6-bp RE is 29.5%, based on a binomial distribution). Many REs will have two or more recognition sites (34.4% probability of multiple recognition sites for 5000-bp fragment); however, some REs are not convenient for cloning purposes because of sensitivity to Dam/Dcm DNA methylation. In addition, with some 6-bp palindromes, no REs with 4-nt overhangs are available.

The overall quantity of the various 6-bp palindromes is 4^3^ = 64, while the overall quantity of the various hybrid recognition sites, including palindromes, is 4^2^ × 4^2^ = 256. The frequency of hybrid recognition sites in a random DNA fragment is one in 4^2^ bp, so the possibility of finding a suitable site within a short region is four times higher. More importantly, if the chosen hybrid site occurs in another part of the target DNA fragment, it will not be rendered unusable, because it will be created after the ligation of the adjacent modules and will not be cut by any RE. The occurrence of recognition sites for both REs, comprising this hybrid site in another part of the target DNA fragment, should not affect the success of the cloning, because only two adjacent modules should be cut by the REs during assembly. The only limitation in using hybrid recognition sites is the presence of the recognition site of the RE from the hybrid recognition site in the same module. The probability of this occurring is only 25% for 1000-bp modules and 12.5% for 500-bp modules.

Use of hybrid restriction sites will greatly simplify MAC. The obvious bottleneck in utilizing such sites, however, is the complexity of identifying their positions in the DNA fragment for generation of a list of possible sub-fragments using the “Find” function in text processors. An option for mapping hybrid recognition sites is not present in the common software packages used for molecular cloning, such as Vector NTI Suite or DNA Star. Hence, a specialized software tool is needed for such calculations.

## Findings

### Algorithm

The task of dividing a given DNA sequence into fragments, restricted by 6-bp palindromes and hybrid recognition sites consists of: 1) generating all possible compatible pairs of REs from the list of those available; 2) searching for 6-bp substrings of generated recognition sites and hybrid sites in the target sequence; and 3) formatting the output of the search results.

### Implementation and program interface

The BIOF program, incorporating this algorithm, is implemented as a Windows-based standalone application using the Delphi programming language [8]. The compiled executable file, samples of data files and the source code are distributed from https://sourceforge.net/projects/biof/files/ under a GNU public license. Delphi compilers are created for many operating systems, and the BIOF source code may be compiled in different program environments.

**Figure 2:**
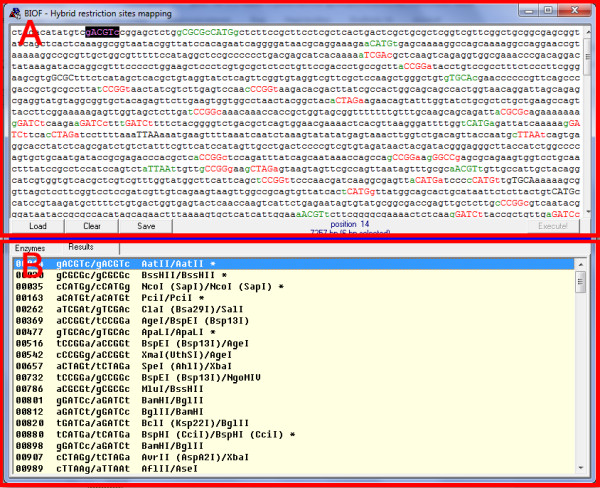
**BIOF program workflow.** In the upper pane (**A**) the DNA sequence being analyzed is displayed. In the lower pane (**B**) the list of imported restriction endonucleases is displayed; this can be switched to the list of hybrid sites found in the DNA sequence.

The BIOF application workflow begins by loading the FASTA-formatted or plain text file with the target DNA sequence (Figure 2). Alternatively, the DNA sequence may be pasted from the clipboard into the upper pane of the application; multiple copy–paste operations and editing of the DNA sequence may be performed within the pane. The application adheres to the FASTA format description [9] and removes non-relevant symbols such as numbers and spaces from the sequence data automatically. Mapping palindromes and hybrid restriction sites starts when the user presses the button “Execute!”. The positions of the sites found by the software are displayed in the lower pane; palindromic restriction sites (i.e. sites that are recognized by one RE) are marked by asterisks. Lists of the sites found are automatically saved in the text file for further use.

The list of available REs used by BIOF for mapping comprises a single Microsoft Excel file with three mandatory fields for each enzyme, i.e., cohesive end sequence, recognition site, and enzyme name. REs are commercially available type II restriction enzymes from REBASE v.112 [10] with a non-ambiguous palindromic hexanucleotide recognition site generating 5’ sticky ends upon cutting. Blunt-end or 3′-cohesive ends generating RE or non-palindromic RE can be added to the list where they will be included in the mapping output, but they will not be used to generate hybrid sites.

The utility of the BIOF program and the MAC strategy was tested with a practical dataset comprising the upstream and downstream non-translated regions of the translation elongation factor 1 alpha gene from Chinese hamster ovary (CHEF1).

## Results and discussion

A genomic region including the CHEF1 gene and its flanking sequences was obtained from a genomic library [11]; the region was sequenced and deposited under the GenBank accession number [GenBank:AY188393]. The authors, Running Deer and Allison [11], found that two fragments of the CHEF1 gene corresponding to positions 8532–12603 and 14545–18794 contained all of the necessary genetic elements for high-level expression of heterologous proteins in cultured mammalian cells. Direct PCR amplification of these fragments using template DNA from cultured Chinese hamster ovary (CHO) DG-44 cells was attempted by our group. No PCR products were obtained that corresponded to these fragments, even after extensive screening to identify optimal annealing temperatures, addition of DMSO or betaine, or alteration of the magnesium ion concentration (data not shown).

Hence the MAC strategy was employed for these two DNA fragments. The upstream (5′-flanking) fragment of the CHEF1 gene was divided into five modules using BIOF (Table 1), and the downstream (3′-flanking) fragment was divided into six modules (Table 2). Three of the nine module junctions contained natural palindromic restriction sites, while six other junctions contained hybrid sites. Most of the modules did not exceed 0.8 kbp in length; therefore, the insert could be fully sequenced on both strands using generic vector-specific primers. All 11 modules were obtained from the DG-44 cell genomic DNA at the first PCR attempt; these were cloned into the T-vector, verified by sequencing multiple plasmid clones, and assembled into two complete DNA fragments using four and five subsequent restriction-ligation procedures, respectively. The resulting fragments were re-sequenced using specific primers and no mutations were found in the assembled modules. It should be noted that two-stranded forward and reverse sequencing of the modules revealed multiple single nucleotide differences when compared with the reference sequence [GenBank:AY188393] and one two-nucleotide insertion, as summarized in Tables 3 and 4. These differences were consistently found in all of the plasmid clone sub-fragments and are supposedly the artifacts of raw data interpretation of the GenBank AY188393 sequence.

**Table 1:** Sub-fragments of the Chinese hamster elongation factor 1 alpha upstream flanking region

**Sub-fragment number**	Start*	End*	**Length**	**5′-flanking RE**	**3′-flanking RE**	Clones sequenced**	**Clones with the correct sequence, %**
1	8532	9117	586	*(XhoI)*	*Avr*II	3	100
2	9114	9800	687	*Xba*I	*Bam*HI	3	67
3	9795	10573	779	*Bam*HI	*Avr*II	3	67
4	10570	11208	639	*Spe*I	*Xho*I	3	100
5	11205	12491	1287	*Sal*I	*(Nco*I*)*	4	100

Nucleotide positions are numbered according to the GenBank entry AY188393. Restriction sites that were used for sub-cloning the assembled fragments into the expression vectors are bracketed.

^*^Start and end positions include the corresponding restriction endonuclease recognition sites.

^**^Following fragment assembly, clones were re-sequenced using specific primers; no mutations were found.

*RE* restriction endonuclease.

**Table 2:** Sub-fragments of the Chinese hamster elongation factor 1 alpha downstream flanking region

**Sub-fragment number**	Start*	End*	**Length**	**5′-flanking RE**	**3′-flanking RE**	Clones sequenced **	**Clones with the correct sequence, %**
1	14545	15157	613	*(Nhe*I*)*	*Xba*I	4	75
2	15154	15908	755	*Nhe*I	*Avr*II	3	100
3	15905	16644	740	*Nhe*I	*Avr*II	3	100
4	16639	17327	689	*Avr*II	*Sac*I	3	100
5	17322	18019	698	*Sac*I	*Bam*HI	4	100
6	18016	18794	780	*Bcl*I	*(Xho*I*)*	5	60

Nucleotide positions are numbered according to GenBank entry AY188393. Restriction sites that were used for sub-cloning the assembled fragments into the expression vectors are bracketed.

^*^Start and end positions include the corresponding restriction endonuclease recognition sites.

^**^Following fragment assembly, clones were re-sequenced using specific primers; no mutations were found.

*RE* restriction endonuclease.

**Table 3:** Differences between the Chinese hamster elongation factor 1 alpha upstream flanking fragments obtained in this study and published data

**#**	**Sub-fragment number**	**Position**	**AFTD01093962**	**Present work**	**AY188393**
1	2	3663-6334	TT	TT	**- -**
					**―**
2	3	4547	A	G	G
3	3	4556	A	G	G
4	3	4561	A	G	G
5	3	4566	A	C	C
6	3	4569-4581	AAAAAGGAGGTGG	GCTA	GCTA
7	4	4978-4979	АС	**- -**	АС
				**―**	
8	4	5256	G	G	-
9	5	5346	G	G	-
10	5	5374	C	C	-
11	5	5396	C	C	-

Nucleotide changes were observed in one of the three aligned sequences (shown underlined). Nucleotide positions are shown according to the GenBank entry AFTD01093962.

**Table 4:** Differences between the Chinese hamster elongation factor 1 alpha downstream flanking fragments obtained in this study and published data

**#**	**Sub-fragment number**	**Position**	**AFTD01093963.1**	**Present work**	**AY188393**
1	1	2349-2353	GTCCC	ATATT	ATATT
2	1	2378	G	C	C
3	1	2510	C	A	A
4	1	2555	A	C	C
5	1	2559	T	A	A
6	1	2587/2588	-	A	A
7	1	2842	C	C	-
8	2	3305	G	G	-
9	2	3674	G	G	C
10	3	3893	T	T	G
11	5	5372	A	G	A
12	6	6160	G	G	-

Nucleotide changes were observed in only one of the three aligned sequences (shown underlined). Nucleotide positions are shown according to the GenBank entry AFTD01093963.1.

After completion of the experimental work presented here, the assembled CHO genome was released [12]; it was found that all except two of the mismatches between our DNA assemblies and the GenBank AY188393 sequence matched our data. It should be noted that the CHO genome shotgun sequencing data, covering the CHEF1 gene [GenBank:AFTD01093962] and [GenBank:AFTD01093963.1], does not completely overlap with the GenBank AY188393 sequence; a 810 bp gap in the shotgun sequencing data spanning from 11387 to 12196 of the GenBank AY188393 sequence exists, hence we compared this fragment with only one published sequence.

At the same time, we found that multiple other nucleotides in the CHO genome assembly data did not match the sequences of our cloned fragments. Most of them are different from both GenBank AY188393 and our own fragments. In two cases (Table 3 line 7 and Table 4 line 11) data from CHO genome assembly and GenBank AY188393 matched each other and differed from our sequence. Presumably, our own sequence data better reflect the true sequence of these regions, because the short cloned DNA modules were sequenced with more redundancy than both the GenBank AY188393 sequence and the CHO genome assembly. In addition, all of the raw data obtained in the present work were analyzed by the researcher, not by machine base-calling procedure that is used for genome-wide shotgun sequencing projects. One single-nucleotide mutation in our fragment and one two-nucleotide insertion that do not match the CHO genome data and the GenBank AY188393 sequence could be PCR-based errors that were present in all of the clones analyzed by us. Alternatively, the mutations might be natural mutations in the CHO DG-44 cell line used by us as the source of genomic DNA. Justification of these possible explanations is beyond the scope of the present work.

The overall cloning strategy employed here has several known alternatives. First of all, direct high-fidelity PCR may be sufficient to clone a desired 5–10-kbp DNA fragment, and successful amplification of a 29.7-kbp DNA fragment has been reported [13]. Self-assembly cloning [14] and similar PCR-based techniques are already developed and may be employed for cloning large DNA fragments. However, although all of them require less effort than MAC, such techniques do not exclude the possibility of modules with mutations in the resulting plasmid.

The cloning strategy presented here may be adopted for creation of cDNAs *in vitro* from genomic DNA, as described in [15]. This technique, which is useful for obtaining ORFs from rare gene transcripts, consists of overlapping PCR with many primers, which bypassed introns. It was tested on the medium-sized polymeric immunoglobulin receptor gene and a mutation rate of 2.5 mutations per clone was found in the 2295 bp product. Implementation of the MAC strategy could, in this case, decrease the proportion of incorrect clones if only target ORF fragments are generated by overlapping PCR, the sequence is verified and only the correct inserts are assembled by restriction-ligation into the full-length ORF.

Pieces of DNA may be assembled together without the need for restriction-ligation reactions. For example, uracil-excision-based cloning allows for ligation-independent cloning of PCR-generated DNA fragments. Such fragments are treated by enzymes, specifically removing the deoxyuracil nucleotide from the PCR primers used, and generating long overhangs that are sufficient for direct bacterial transformation of the resulting linear DNA mixture [16]. This technique may be used instead of the MAC strategy proposed by us, but all PCR-generated DNA sub-fragments must be annealed together and cloned in one step, greatly decreasing the chance of obtaining a plasmid clone without PCR-generated mutations.

## Conclusions

The MAC technique, aided by the specialized BIOF program tool, allows high precision cloning of long DNA fragments from genomic DNA. This cloning strategy may be easily adopted for creation of artificial DNA fragments, such as spliced cDNAs. Crucially, MAC eliminates the necessity to construct DNA libraries from genomic DNA.

## Materials and Methods

Genomic DNA was purified from CHO DG-44 cells (Invitrogen, Carlsbad, CA) using a Genomic DNA Purification Kit (#K0512 Fermentas, Vilnius, Lithuania). Oligonucleotides were synthesized by Evrogen JSC (Moscow, Russia). PCR mixes were prepared using an Encyclo PCR kit or Taq-polymerase (#PK001 or #PK013 Evrogen, Moscow, Russia) according to the manufacturer’s instructions. A PTC-100 Thermal Cycler (Bio-Rad Laboratories, Hercules, CA) was used for PCR. The PCR program consisted of a denaturation step cycle at 95°C for 3–5 min, 35 cycles at 95°C for 15 s, 55–58°C for 20 s, 72°C for 1 min and a final elongation cycle at 72°C for 5 min. In the case of fragment 5 of the CHEF1-1a 5′flanking region, 5% DMSO was added to the PCR mixture.

PCR products were purified from 1% agarose gels or from PCR samples using the Wizard SV Gel and PCR Clean-Up System (#A9282 Promega, Madison, WI). PCR products were cloned into pAL-TA (#TA001 Evrogen, Moscow, Russia) or pGEM-T (#A3600 Promega, Madison, WI) vectors using T4 DNA ligase (#EL0011 Fermentas, Vilnius, Lithuania), before being used to transform the *Escherichia coli* TOP10 strain (Invitrogen, Carlsbad, CA). Recombinants, selected by the blue/white screening, were picked and grown overnight at 37°C in Luria broth with ampicillin. Cells from overnight cultures were collected by centrifugation and plasmids were isolated using a GeneJet Plasmid Purification Kit (#K0503 Fermentas, Vilnius, Lithuania), then sequenced. Sequencing reactions were performed using the BigDye Terminator, version 3.1 sequencing kit (Applied Biosystems, Foster City, CA) and run on an ABI PRISM 3730 genetic analyzer (Applied Biosystems). Sequence data were edited using Chromas 1.45 (Technelysium Pty Ltd., Australia). Database searches of GenBank were performed using the BLASTN algorithm.

DNA fragment assembly was performed with the restriction enzymes *Xho*I, *Xba*I, *BamH*I, *Sal*I, *EcoR*I, *Nhe*I, *Bcl*I (Fermentas, Vilnius, Lithuania), or *Ahl*I (isoschizomer *Spe*I), *Psp124B*I (isoschizomer *Sac*I), *Ksp22*I (isoschizomer *Bcl*I), *AspA2*I (isoschizomer *Avr*II) (Sibenzyme, Novosibirsk, Russia).

## Availability and requirements

▪ **Project name:** BIOF

▪ **Project home page:**http://sourceforge.net/projects/biof/

▪ **Operating system(s):** Windows 2000 and above

▪ **Programming language:** Delphi 7

▪ **Other requirements:** none

▪ **License:** GNU GPL

## Abbreviations

PCR: Polymerase chain reaction; Kbp: Kilo base pairs (1000 base pairs); MAC: Modular assembly cloning; cDNA: Complementary DNA; DMSO: Dimethyl sulfoxide; RE: Restriction endonuclease; CHEF1: Chinese hamster Elongation factor 1 alpha.

## Competing interests

The authors declare that they have no competing interests.

## Authors’ contributions

NAO developed the experimental strategy, performed the BIOF program testing and implementation, performed cloning procedures, and drafted the manuscript. AVO wrote the BIOF program. IIV initiated the project, participated in its design and coordination, and helped to draft the manuscript. All of the authors have read and approved the final manuscript.
